# Herbicide cycling has diverse effects on evolution of resistance in *Chlamydomonas reinhardtii*

**DOI:** 10.1111/j.1752-4571.2012.00276.x

**Published:** 2012-06-11

**Authors:** Mato Lagator, Tom Vogwill, Nick Colegrave, Paul Neve

**Affiliations:** 1 School of Life Sciences, University of WarwickCoventry, UK; 2 School of Biological Sciences, Institute of Evolutionary Biology, University of EdinburghEdinburgh, UK

**Keywords:** *Chlamydomonas reinhardtii*, cross-resistance, experimental evolution, fitness costs, herbicide resistance, herbicide rotation

## Abstract

Cycling pesticides has been proposed as a means of retarding the evolution of resistance, but its efficacy has rarely been empirically tested. We evolved populations of *Chlamydomonas reinhardtii* in the presence of three herbicides: atrazine, glyphosate and carbetamide. Populations were exposed to a weekly, biweekly and triweekly cycling between all three pairwise combinations of herbicides and continuously to each of the three herbicides. We explored the impacts of herbicide cycling on the rate of resistance evolution, the level of resistance selected, the cost of resistance and the degree of generality (cross-resistance) observed. Herbicide cycling resulted in a diversity of outcomes: preventing evolution of resistance for some combinations of herbicides, having no impacts for others and increasing rates of resistance evolution in some instances. Weekly cycling of atrazine and carbetamide resulted in selection of a generalist population. This population had a higher level of resistance, and this generalist resistance was associated with a cost. The level of resistance selected did not vary amongst other regimes. Costs of resistance were generally highest when cycling was more frequent. Our data suggest that the effects of herbicide cycling on the evolution of resistance may be more complex and less favourable than generally assumed.

## Introduction

Synthetic herbicides have become the dominant means of controlling weedy plants in agricultural settings (Powles and Shaner 2001), and evolution of resistance to herbicides is widespread (Heap 2011). In general terms, there are two modes of herbicide resistance evolution: target-site resistance and non-target-site resistance (reviewed in Powles and Yu 2010). Target-site resistance confers resistance to a single herbicide mode of action, whereas non-target-site resistance may result in complex patterns of cross-resistance rendering populations resistant to multiple modes of action (Powles and Yu 2010). In evolutionary terms, target-site and non-target-site resistance represent specialist and generalist modes of herbicide resistance, respectively. As both mechanisms can provide resistance to the same herbicide, specialist and generalist phenotypes can coexist.

A key challenge in herbicide, as well as pesticide and antibiotic resistance research, is to design management strategies that effectively deploy a range of modes of action to retard or prevent evolution of resistance (Georghiou and Taylor 1986; Powles and Yu 2010). A commonly recommended practice is to cycle chemicals with different modes of action (Beckie 2006). Cycling (often referred to as herbicide rotation) introduces temporal environmental heterogeneity so that sequential generations are exposed to different selection pressures. This can potentially affect the rate of resistance evolution in a number of ways. First, over a given time scale, fewer generations are exposed to any single environment, leading to reduced selection for resistance to each component environment (MacArthur 1964; Futuyma and Moreno 1988; Whitlock 1996). Second, if adaptation to one environment incurs a fitness cost in others, cycling may retard or even prevent resistance evolution (Leeper et al. 1986; Gressel and Segel 1990). Additionally, environments in which herbicides are cycled are more complex and may require a greater degree of genetic variation for adaptation to occur. However, ecological and evolutionary theory would predict that environments characterized by a greater degree of temporal heterogeneity would result in the evolution of more generalist phenotypes (Gavrilets and Scheiner 1993; Chesson 2000; Kassen 2002), and hence it may also be the case that cycling exacerbates the spread of generalist resistance phenotypes (Gomulkiewicz and Kirkpatrick 1992; Tufto 2000). This effect is therefore likely to crucially depend on the frequency of cycling between different modes of action, with more rapid rates of switching more strongly favouring generalist types of resistance.

The difficulties associated with performing selection experiments on large weed populations with slow generation times (one generation per year) have limited the testing of these hypotheses mostly to theoretical and simulation models, with only a few experimental studies (Porcher et al. 2004; Roux et al. 2005; Kover et al. 2009; Springate et al. 2011). Models have shown that, in the absence of pleiotropic costs of resistance, cycling may not retard resistance evolution (Diggle et al. 2003; Bergstrom et al. 2004; Roux et al. 2008). It is not possible to generalize on the existence of pleiotropic costs associated with evolved resistance to herbicides, as it seems that fitness costs vary according to the mechanism of resistance (reviewed by Vila-Aiub et al. 2009). A similar lack of understanding of the dynamics of resistance evolution has led to failed attempts to slow the spread of resistance to antibiotics in clinical settings (Bergstrom and Feldgarden 2007).

The techniques of microbial experimental evolution can be applied to a range of fundamental and more applied evolutionary questions (Buckling et al. 2009), including the evolution of resistance to antimicrobials (Perron et al. 2008; Hall et al. 2010; MacLean et al. 2010). Experimental evolution with *Chlamydomonas reinhardtii* offers the potential to better understand the evolution of resistance to herbicides and to experimentally test resistance management strategies. *Chlamydomonas reinhardtii* is a unicellular green chlorophyte, capable of growing as a photoautotroph and a heterotroph. Under laboratory conditions it grows asexually (Harris 2008). It is susceptible to a range of commercial herbicides (Reboud et al. 2007). In the current study, we experimentally evolved populations of *C. reinhardtii* with sequential cycling between pairwise combinations of three herbicides with different modes of action: glyphosate, atrazine and carbetamide. The frequency of cycling between herbicides was varied to explore the impacts of the degree of environmental heterogeneity on the dynamics of resistance evolution. In particular, we were interested in investigating if (i) cycling leads to reduced rates of resistance evolution, (ii) there was a relationship between the frequency of cycling and the rates and outcomes of evolution, (iii) cycling leads to comparable levels of resistance as homogeneous environments and (iv) cycling could result in the selection of more generalist resistance phenotypes.

## Methods and materials

### Founding population

*Chlamydomonas reinhardtii* CC-1690, a wild-type positive mating strain obtained from the *Chlamydomonas* Resource Center's core collection, was used in this experiment. Prior to selection experiments, the strain had been adapted to liquid Bold's medium through continuous exposure for over 700 generations. Two weeks before the start of selection procedure, 20 μL of the founding population (approximately 15 000 cells) was spread on an agar plate. After 7 days of growth, a single colony was picked and used to inoculate a Bold's medium liquid culture. This colony was multiplied for 7 days and was used to found all experimentally evolving populations.

### Culture conditions

The culture medium used in all experimental conditions is modified Bold's Medium (subsequently BM) (Colegrave et al. 2002). Populations were grown in disposable borosilicate glass 25 × 150 mm tubes, in 20 mL of BM and maintained in an orbital shaker incubator, at 28°C and 180 rpm, under continuous light exposure, provided by six fluorescent tubes mounted in the incubator lid (Osram L30 W/21-840, cool white; light intensity at the location of the tubes was 161 μmol m^−2^s^−1^). Cultures were transferred into fresh BM every 7 days (see below), during which time the ancestral population growing in the absence of herbicides would have reached stationary phase (3.1 × 10^7^ cells).

### Herbicides

We exposed populations to three herbicides: atrazine, glyphosate and carbetamide. The herbicides have different modes of action (atrazine, photosystem II inhibitor; glyphosate, inhibitor of aromatic amino acid synthesis and carbutamide, mitosis inhibitor). Prior to the selection procedure, we determined the minimum inhibitory concentration (MIC) of each herbicide, this being the minimum concentration that prevented detectable population growth over 4 days.

### Cycling regimes

Three experimental conditions involved continuous exposure to a single herbicide (A0 denoting continuous exposure to atrazine, G0 to glyphosate and C0 to carbetamide). A weekly, biweekly and triweekly cycling regime was created for all three possible pairwise combinations of herbicides (AG1 denoting the weekly cycle between atrazine and glyphosate, AG2 the biweekly cycle and so on). Each experimental condition (12 in total) was replicated 6 times, giving rise to 72 independently evolving populations. Six populations were propagated by serial transfer in the absence of herbicides. Throughout the experiment one of these six ‘source’ populations provided immigration into each of the six replicate treatments as required (see below). These populations also acted as controls.

Approximately 125 000 cells (estimated by absorbance at 750 nm) from the founding population provided the initial population for each of the 78 populations. At each transfer, 200 μL of the evolving culture was transferred into fresh media. If the number of cells in 200 μL of culture medium was estimated to be <125 000, as would happen until resistance was developed, then the appropriate number of cells from one of the source populations was added to make the total cell number at the transfer approximately 125 000. Therefore, the minimum number of cells at the beginning of each cycle was 125 000. For each of the six replicates, the same source population was used for immigration throughout the experiment. According to this protocol, when undergoing sufficient growth (at least 6.64 cell division in 7 days), a population is under soft selection and capable of maintaining itself after the weekly bottleneck event. When growth did not reach this number of cell divisions, weekly bottlenecks would drive the population towards extinction, and these populations were maintained by immigration from the corresponding source population. The experiment was carried out for 12 transfer cycles (12 weeks).

### Measuring the rates of evolution

The optical density at 750 nm (OD_750_) was measured in a Jenway 6315 benchtop-spectrophotometer 4 days after the transfer. OD_750_ was converted into population size using a calibration curve obtained by correlating OD_750_ with cell counts in 70 independent samples. Resistance was considered to have evolved when detectable population growth was consistently measured (OD_750_ > 0.045, corresponding to at least three cell divisions). The rate of resistance evolution was quantified by measuring the first week when resistance was observed. The rate of resistance evolution to each component herbicide in cycling regimes was expressed as the number of weeks that the population had been exposed to that herbicide.

### Isolation of the evolved populations

In order to ensure that populations used for subsequent resistance and fitness assays contained only herbicide-resistant cells, approximately 20 000 cells of each final population were plated on BM agar plates that contained the MIC of a single herbicide. For cycling regimes, 20 000 cells of each final population were plated independently onto two plates, one containing each of the herbicides that the population had been exposed to. After 7 days of growth, 200 colonies from each population were randomly selected and used to inoculate a fresh population in liquid BM. If the population had been exposed to two herbicides, 100 colonies were randomly selected from each of the plates containing those herbicides and used to inoculate a fresh population in liquid BM. These populations were grown for 7 days prior to conducting further assays. In addition, for lines evolving under cycling regimes, 10 single colonies from each BM + herbicide plate were picked and multiplied for 7 days in BM. For all 10 populations, 125 000 cells were then transferred into MIC of the second herbicide from that cycling regime. In all cases, populations derived from single cells were resistant to both herbicides in the cycling regime, indicating that evolved populations always consisted of individuals with resistance to both herbicides cycled, rather than to mixtures of individuals with resistance to individual cycle components.

### Level of resistance and fitness in the ancestral environment

The growth rate of the evolved populations in the selective environments (hereinafter ‘level of resistance’) was determined by measuring population growth in liquid culture at the MIC of both herbicides to which that population had been exposed. A total of 125 000 cells were used to inoculate each resistance assay, and population size was determined by measuring optical density at 750 nm after 4 days growth. This was replicated twice for each evolved population, and the mean number of cell divisions completed during 4 days growth was used as an estimate of the level of resistance. In order to assess if adaptation to herbicide environments was associated with a fitness cost, the comparative growth rate of evolved and source populations in the herbicide-free environment was estimated. This measure was replicated twice for each evolved population and the mean number of cell divisions completed during 4 days growth was used as an estimate of fitness in the ancestral environment. Both levels of resistance and fitness in the ancestral environment were expressed as a proportion of the growth of source populations in the ancestral (BM only) environment.

### The degree of generality

To test for cross-resistance, we assayed the growth of evolved populations at the MIC of four herbicides to which they had no previous exposure (tembotrione, iodosulfuron-methyl-sodium, isoproturon and *S*-metolachlor), as well as whichever of atrazine, glyphosate or carbetamide they had not been exposed to (i.e. we also assayed cross-resistance to carbetamide in populations evolved in cycling between atrazine and glyphosate). A total of 125 000 cells of the evolved populations were inoculated into tubes containing one of these herbicides and population growth was measured after 4 days. If growth was significantly different from that of the source populations under the same conditions, the population was deemed to have evolved cross-resistance. Each condition was replicated twice.

### Cross-protection assays

To investigate a possible contribution of cross-protection, the phenomenon whereby exposure to one stress provides a degree of physiological acclimation (cross-protection) to subsequent stresses, we grew naïve *C. reinhardtii* populations in the presence of low doses (0.8 MIC for atrazine, 0.7 MIC for glyphosate and carbetamide) of each of our three herbicides. Doses below MIC were used so that detectable population growth was apparent between transfer periods. After 7 days in one herbicide we transferred 125 000 cells into below MIC doses of each of the two other herbicides. We also transferred 125 000 cells without previous herbicide exposure into below MIC doses of all three herbicides as a control. Four days after transfer, growth rates of each population were estimated. Each condition was replicated three times.

### Statistical analysis

The rate of resistance evolution (weeks to resistance) was analyzed using a Cox regression. The herbicide regime was fitted as a covariate, with the ancestral immigration source as the strata. For cycling regimes, the number of weeks until resistance evolved to individual herbicide components (weeks exposed to that herbicide) were compared to rates of evolution of resistance when continuously exposed to that herbicide. The Cox regressions were performed in SPSS. The level of resistance and fitness in the ancestral environments of the evolved populations were first analyzed using a General Linear Model with the herbicide cycled and the cycling frequency as fixed factors, and ancestral immigration source as the random factor. We also investigated the interaction between herbicide and cycling frequency. When populations under a cycling regime evolved resistance to only one of the herbicides, we only analyzed the effects of the cycling frequency, making it a fixed factor. The level of resistance to individual herbicide in cycling regimes was subsequently compared to resistance in the continuous exposure treatment using a Dunnett's corrected paired *t*-test, with the herbicide regime fitted as a fixed factor, and the ancestral immigration source as the random factor. When some populations in a regime did not evolve resistance, we compared them to the continuous exposure treatment using a Dunnett's corrected *t*-test. The level of resistance of the three continuous exposure populations was compared in the same fashion. The fitness in the ancestral environment of all populations was compared to source populations and to populations that underwent continuous exposure using a Dunnett's corrected paired *t*-test, except when some of the populations in a regime did not evolve resistance, in which case Dunnett's corrected *t*-test was used. The fitness in the ancestral environment of the three continuous exposure regimes was compared in the same fashion. Growth rates from the cross-protection assay were compared between the populations that underwent previous exposure to an herbicide and those that did not in a Dunnett's corrected paired *t*-test. The previous herbicide the population was exposed to was fitted as a fixed factor, and the replicate population as the random factor.

## Results

### Dynamics of herbicide resistance

**Figure 1:**
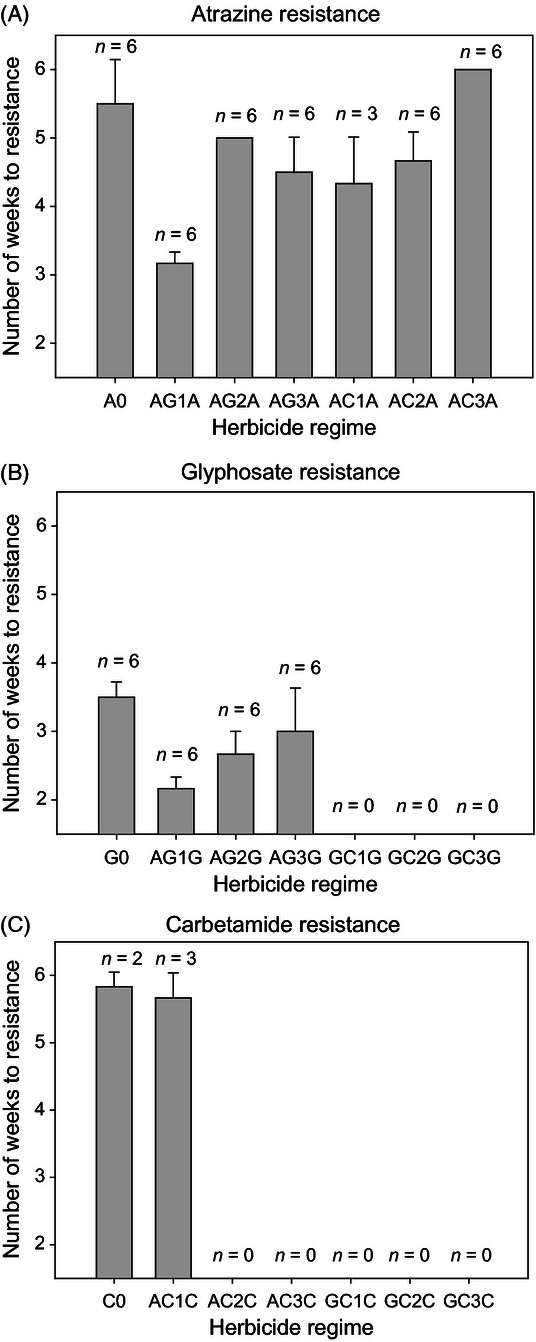
The dynamics of resistance evolution measured as number of weeks until resistance evolved. Bars represent the mean weeks to resistance amongst the replicates where resistance was observed; *n* is the number of replicate populations that evolved resistance: (A) atrazine resistance (A0 indicates continuous exposure to atrazine, AG1, AG2, AG3 a weekly, biweekly and triweekly rotation between atrazine and glyphosate, respectively. AC1, AC2 and AC3 refer to weekly, biweekly and triweekly rotation between atrazine and carbetamide, respectively); (B) glyphosate resistance (labelling convention as above) and (C) carbetamide resistance. Error bars are standard errors of the mean.

Evolution of herbicide resistance was observed in many populations, under various continuous exposure and cycling regimes. Resistance evolved in all populations with continuous exposure to atrazine (Fig. 1A) or glyphosate (Fig. 1B), and to both herbicides in all populations that underwent cycling between these two herbicides (Fig. 1A,B). Resistance evolved in two of six populations that underwent continuous carbetamide exposure (Fig. 1C), while resistance to both atrazine and carbetamide evolved in three of six populations that underwent weekly cycling between the two (Fig. 1A,C). Atrazine, but not carbetamide resistance, evolved in all populations under a bi- and triweekly cycle between the two herbicides (Fig. 1A,C). No resistant individuals were observed in the populations cycling between glyphosate and carbetamide (Fig. 1B,C). These results demonstrate that cycling can prevent, accelerate or have no impact on the evolution of resistance to herbicides.

Continuous exposure to glyphosate resulted in significantly more rapid evolution of resistance than continuous exposure to atrazine (*z* = 6.096, *P* < 0.05) or carbetamide (*z* = 6.083, *P* < 0.05). Rates of evolution of atrazine and carbetamide resistance were not significantly different.

The number of weeks until resistance evolved to individual herbicides in cycling regimes was compared for each regime to the rate of evolution in populations that underwent continuous exposure to that herbicide. Resistance to atrazine evolved more rapidly in a weekly cycle between atrazine and glyphosate (*z* = 10.169, *P* = 0.001) (Fig. 1A). Though there was a trend towards more rapid evolution of atrazine resistance in the biweekly (*z* = 3.381, *P* = 0.066) and triweekly cycle with glyphosate (*z* = 3.369, *P* = 0.066), these differences were not significant (Fig. 1A). A weekly cycle between atrazine and glyphosate yielded faster-evolving resistance to glyphosate than continuous exposure to glyphosate (*z* = 3.930, *P* = 0.047) (Fig. 1B). Rates of evolution of carbetamide resistance were not significantly different between any of the regimes in which it evolved.

### Level of resistance

We express the level of resistance as the proportion of growth rate retained in populations with evolved resistance in comparison to source populations in herbicide-free environments. In continuous selection regimes, the level of resistance was greater in populations exposed to glyphosate than in atrazine-resistant (*T*_10_ = 19.61, *P* < 0.01) and carbetamide-resistant populations (*T*_6_ = 5.963, *P* < 0.005). Carbetamide-resistant populations had a higher level of resistance than atrazine-resistant populations (*T*_6_ = 4.854, *P* < 0.01).

**Figure 2:**
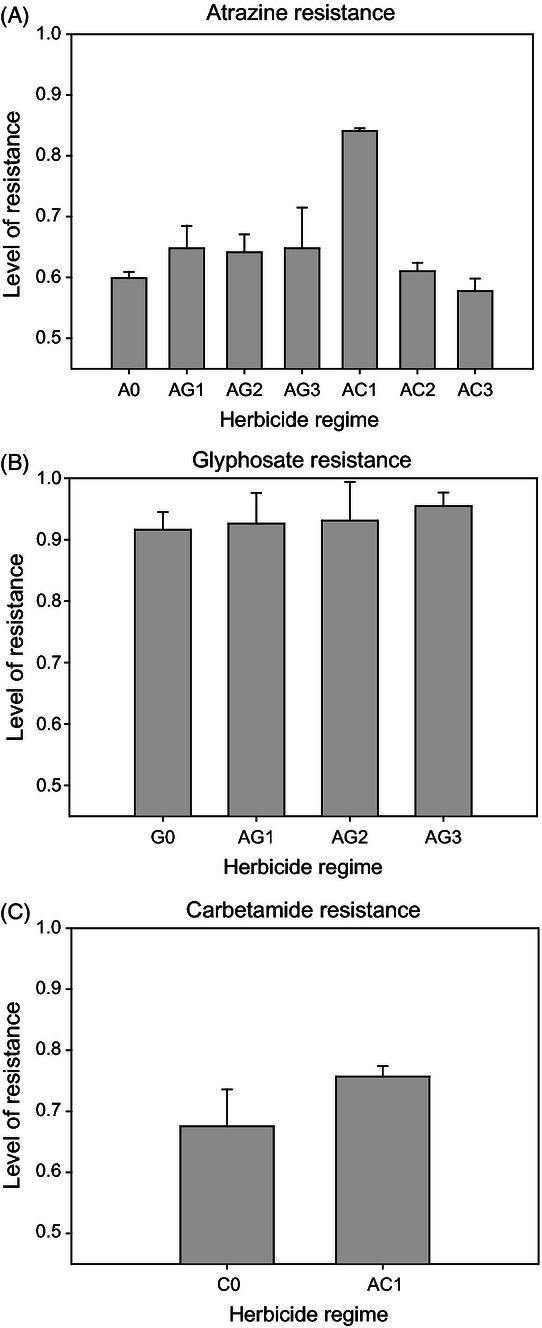
The level of evolved resistance expressed as the proportion of growth retained in herbicide environments in comparison with source populations in herbicide-free environments. Bars are mean values of all the evolved replicates in each condition: (A) atrazine level of resistance, (B) glyphosate level of resistance and (C) carbetamide level of resistance. Error bars are standard errors of the mean.

Overall, in cycling regimes, the herbicide that atrazine was cycled with had no significant impact on the level of atrazine resistance. However, the frequency of cycling did significantly affect the level of resistance (*F*_2,16_ = 8.10, *P* < 0.005), and there was a significant interaction between the herbicide used and the frequency of cycling (*F*_2,16_ = 8.03, *P* < 0.005). As indicated by Dunnett's corrected *t*-tests, the levels of atrazine resistance that evolved in the AC1 regime were significantly greater than in continuous atrazine exposure regimes (*T*_7_ = 5.487, *P* < 0.001), as well as all other regimes (Fig. 2A). For glyphosate and carbetamide resistance there were no significant differences in the level of evolved resistance in any of the regimes in which resistance evolved (Fig. 2B,C).

### Fitness in the ancestral environment

**Figure 3:**
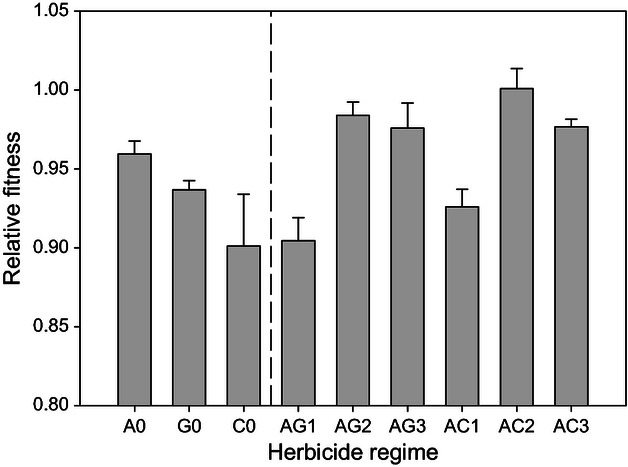
Growth rates in the absence of herbicides of populations with evolved resistance expressed as the proportion of the source populations' growth rate in herbicide-free environments. Bars are mean values of all the evolved replicates in each condition. Error bars are standard errors of the mean.

Comparing the fitness in the ancestral environment (defined as the relative growth rate of evolved populations in BM) of evolved populations to the source populations, we found fitness costs (a significant difference between growth rate in BM of the ancestral and evolved populations) to be frequently associated with evolved resistance (Fig. 3). All populations that evolved resistance in continuous exposure to a single herbicide exhibited a significant reduction in fitness in the ancestral environment – exposure to atrazine (*T*_10_ = −2.80, *P* < 0.05), glyphosate (*T*_10_ = −9.76, *P* < 0.001) and carbetamide (*T*_6_ = −4.711, *P* < 0.05) (Fig. 3). The fitness in the ancestral environment of populations evolved under continuous exposure to atrazine was significantly higher than in the populations evolved in continuous exposure to glyphosate (*T*_10_ = 3.95, *P* < 0.01) or carbetamide (*T*_6_ = 3.598, *P* < 0.05). Reduced fitness in the ancestral environment was also observed in populations under weekly cycle between atrazine and glyphosate (*T*_10_ = −5.94, *P* < 0.001) and weekly cycle between atrazine and carbetamide (*T*_7_ = −6.034, *P* < 0.001) (Fig. 3). Populations that evolved in a bi- and triweekly cycle between atrazine and glyphosate or atrazine and carbetamide did not exhibit significant fitness costs.

### Cross-resistance

**Figure 4:**
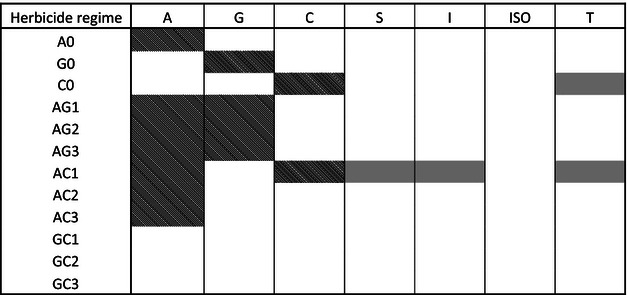
Resistance profiles for evolved populations. Hatched shading indicates resistance to herbicides included in corresponding selection regimes. Cross-resistance to herbicides to which populations had no previous exposure is indicated by grey shading. Cross-resistance was only selected in the C0 and AC1 regimes. A = atrazine; G = glyphosate; C = carbetamide; S = *S*-metolachlor; I = iodosulphuron; Iso = isoproturon; T = tembotrione.

For most selection regimes, no cross-resistance was observed (Fig. 4). Only populations selected under a weekly cycle between atrazine and carbetamide and under continuous exposure to carbetamide exhibited cross-resistance to herbicides to which they had never been exposed (Fig. 4). All of these populations exhibited growth at the MIC of the herbicide tembotrione. All three populations that evolved resistance to both atrazine and carbetamide under a weekly cycle were also resistant to *S*-metolachlor and iodosulfuron.

### Cross-protection

**Figure 5:**
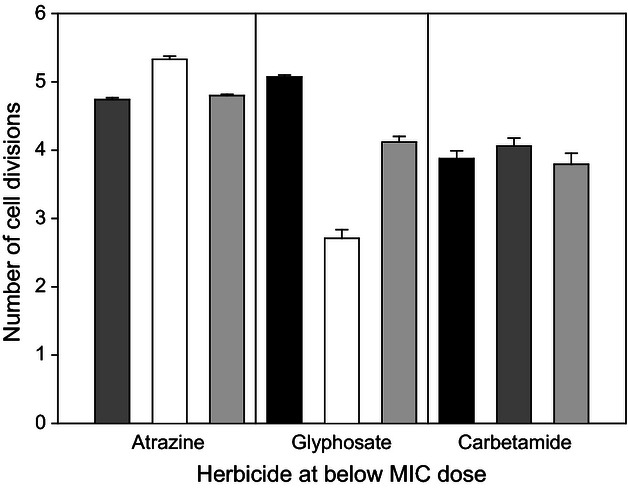
Cross protection. Number of cell divisions the populations underwent after 4 days in below MIC levels of the indicated herbicide. Bars represent mean values. Black bars indicate the populations with previous exposure to atrazine, dark grey bars previous exposure to glyphosate, white bars previous exposure to carbetamide and light grey bars indicate the populations with no previous herbicide exposure. Error bars are standard errors of the mean.

Seven days of exposure to carbetamide significantly increased the growth rates in 0.8 MIC of atrazine when compared to the populations that had no previous exposure to any herbicides (Fig. 5) (*T*_4_ = 7.801, *P* < 0.005). Previous exposure to atrazine significantly increased the growth rates in glyphosate (*T*_4_ = 7.64, *P* < 0.005), while the exposure to carbetamide decreased subsequent growth rates in glyphosate (*T*_4_ = −5.732, *P* < 0.01).

## Discussion

In spite of the lack of evidence for its effectiveness, herbicide cycling has been advocated as a means of slowing or preventing evolution of herbicide resistance (Beckie 2006). A successful cycling strategy must do more than simply extend the chronological time until resistance evolves as this outcome will result simply from the fact that the population is exposed to each component herbicide for less time. A truly effective strategy must increase the time that a population can be exposed (selection time) to, at least one of the cycled herbicides before resistance evolves. In other words, if continuous exposure to herbicide *A* results in evolution of resistance in selection time *x* and continuous exposure to herbicide *B* results in resistance in selection time *y*, when *A* and *B* are cycled, the strategy is successful if either *x*, *y* or the sum of *x* and *y* is increased. According to these criteria, in this study, we have shown that cycling between pairwise combinations of three herbicides can slow, accelerate or have no impact on the dynamics of selection for herbicide resistance. These contrasting outcomes depend on the herbicides being cycled and the frequency of cycling.

### Dynamics of resistance under herbicide cycling

Fitness costs associated with resistance are seen as key determinants of the effectiveness of cycling (Leeper et al. 1986; Gressel and Segel 1990; Jasieniuk et al. 1996). In our study, fitness costs (significantly lower growth rates in absence of herbicide) were not universally observed as found in other studies (McCart et al. 2005; Lopes et al. 2008). Models assuming no fitness costs have predicted that cycling will be ineffective in slowing down the evolution of resistance in selection time (Diggle et al. 2003; Neve 2008). Our results support this general trend, as cycling was most effective when occurring between herbicides where evolved resistance yielded the highest cost (glyphosate and carbetamide) and was much less effective when less costly atrazine resistance evolved (Fig. 1).

It seems somewhat counterintuitive that cycling regimes can, in some instances, increase rates of resistance evolution. We offer two explanations: (i) cross-protection, and (ii) population size effects that can account for increased rates of glyphosate and atrazine resistance evolution, respectively, in the AG regimes. Cross-protection gives rise to a temporary increase in growth rates in one stressful environment after exposure to another (Hill et al. 2002), and a variety of sublethal stresses have been shown to alter antibiotic resistance evolution (McMahon et al. 2006). We have found that exposure to atrazine offers positive cross-protection to glyphosate (Fig. 5) and hypothesize that this phenomenon accounts for enhanced rates of glyphosate resistance evolution in the weekly atrazine and glyphosate cycling regime, as it increases the number of non-resistant cells replicating in glyphosate, increasing population size and mutation supply rate. Assuming that cross-protection is a transient effect, this hypothesis is supported by the observation that increased rates of glyphosate resistance evolution are only observed in the weekly cycle. In relation to increased rates of atrazine resistance evolution in the AG1 regime we conclude that increases in population size, driven by the relatively rapid evolution of glyphosate resistance, are resulting in an increased probability of atrazine-resistant mutations arising in the glyphosate-resistant background. Once this occurs, atrazine resistance is selected in both phases of the cycling regime and hence evolution of atrazine resistance (measured in selection-time) is accelerated. We predict that this dynamic is likely to occur when rapid cycling occurs between pesticides where the rate of resistance evolution varies substantially.

### Impacts of cycling on the evolution of generalists

The frequency of cycling has the potential to change the trajectory of evolution as evidenced by the evolution of a generalist phenotype in the weekly atrazine and carbetamide cycle and no evolution of resistance in bi- and triweekly cycles. Even though this generalist phenotype conferred significantly higher levels of atrazine resistance, it was never selected in the continuous atrazine regime. A number of explanations are possible here. It may be that the generalist phenotype requires fixation of more than one mutation and that the initial mutation confers low levels of resistance to atrazine and carbetamide while carrying a high fitness cost. In a weekly cycle, populations are exposed to carbetamide frequently enough that these mutations are maintained, whereas in other regimes with more frequent or lengthier periods of exposure to atrazine, they are lost due to clonal interference and population bottlenecks. It could also be that the first mutations that get fixed in the population affect the fitness consequences of others, as reported for antibiotic resistance (Trindade et al. 2009; Yeh et al. 2009). Indeed, if the fixation of mutations that confer resistance to atrazine modify the genetic background such that subsequent mutations conferring resistance to carbetamide have a higher selection coefficient (positive epistasis), then generalists are more likely to evolve in a cyclic environment compared to a homogeneous one. In general, it appears that the outcomes of herbicide selection regimes are contingent on complex interactions between the level of resistance, costs of resistance, frequencies of different mutations, cross-resistance phenotypes and the scale of temporal heterogeneity.

In the pesticide and antibiotic literature, generalist resistance usually refers to single mechanisms that confer resistance to multiple toxin modes of action (Delye et al. 2011; for multidrug antibiotic resistance – Alekshun and Levy 2007). The expectation is that generalism will confer lower levels of resistance, often at a higher cost and will therefore only be selected in environments with spatial or temporal variation in selection pressures (Georghiou and Taylor 1986; Futuyma and Moreno 1988; Kassen 2002; Gressel 2009). In this study, broad generalist resistance was selected in the weekly cycle between atrazine and carbetamide, providing some evidence that cycling promotes the evolution of generalist resistance, though in most cycling regimes generalist phenotypes were not observed. Contrary to the major theoretical (Via and Lande 1985; Ravigné et al. 2009), most experimental (Morgan et al. 2009; Legros and Koella 2010; Hall et al. 2010) and the findings in pesticide-resistant organisms (Gressel 2002; Jonsson et al. 2010), we found generalists to have a significantly higher resistance than specialists in both of the selective environments, as well as comparable growth rates in absence of herbicides to the specialists (populations that underwent continuous exposure), a result previously reported for other traits (Turner and Elena 2000; Buckling et al. 2007).

### Cycling affects fitness costs

The accumulation of multiple discrete mechanisms of resistance is an alternative means via which a more generalist resistance phenotype may evolve, and it seems likely that this accounts for evolved resistance to atrazine and glyphosate in the atrazine and glyphosate cycling regimes. The evolution of this multiple resistance may be constrained by the accumulation of fitness costs associated with each resistance trait, particularly where these costs are additive, or potentially even synergistic. In populations that evolved resistance in a weekly atrazine and glyphosate cycle, the growth rates in absence of herbicide are significantly lower than in continuous exposure to atrazine and seem to be additive (Fig. 3), suggesting there may be a limit to multiple resistance in the absence of compensations (Andersson and Hughes 2010; Hall et al. 2010). Bi- and triweekly cycles between atrazine and glyphosate resulted in significantly higher growth rates in the absence of herbicides (Fig. 3) than the weekly cycle or continuous exposure to either herbicide. It therefore appears that lower frequencies of cycling favour the compensation of fitness costs as longer periods spent in the non-focal environment will favour selection for reduced costs of resistance. Alternatively, more heterogeneous environments have lower chance of leading to a global optimum (Collins 2011), and as such less rapid rates of cycling could be more effectively selected for mutations with lower fitness cost.

### Herbicide cycling: forward with caution

Herbicide cycling has been advocated for resistance management as it introduces environmental complexity and heterogeneity and thus may slow adaptation. Results from this study illustrate that cycling can result in diverse outcomes, though some caution is advisable in translating results to annual weedy plants. Temporal heterogeneity of environments may impact the direction of evolution (Levins 1968; Kassen and Bell 1998; Jasmin and Kassen 2007; Venail et al. 2011), with more fine-grained environments (where environment varies at a rate faster than the generation time) favouring more generalist traits. In our design even the rapid rates of cycling far exceeded the generation time of *C. reinhardtii*, meaning that all the environments were coarse-grained. The herbicide cycling advocated for weed management is fine-grained, generally requiring alternating generations to be exposed to different herbicide modes of action. In addition, the order in which the herbicides are cycled could affect the trajectory of evolution and this was not explored. *Chlamydomonas* is haploid and reproduction in these experiments was asexual. Higher plants have complex and diverse modes of sexual and asexual reproduction. There may also be gene flow between evolving meta-populations of agricultural weeds. Finally, most annual weedy plants have a soil reservoir of dormant seeds that acts as a temporal refuge from herbicide selection. Notwithstanding these important differences, our results clearly demonstrate that herbicide cycling may not always slow the rate of evolution of resistance and may result in the evolution of generalist resistance phenotypes resistant to a broad range of herbicide modes of action.

## Data archiving statement

Data for this study are provided in the Supporting information.

## Supporting Information

**Data S1.** Raw experimental data
